# Advancing wound healing: controlled release of tannic acid via epitope imprinted antimicrobial spongy cover material

**DOI:** 10.1007/s11274-025-04266-1

**Published:** 2025-02-04

**Authors:** Büşra Tuna, Pırıl Arısoy, Hatice İmge Oktay Başeğmez, Gözde Baydemir Peşint

**Affiliations:** Department of Bioengineering, Adana Alparslan Türkeş Science and Technology University, Sarıçam, 01250 Adana, Türkiye

**Keywords:** Controlled release systems, Epitope-imprinted polymers, Spongy cover materials, Tannic acid, Wound healing

## Abstract

The increasing resistance of microorganisms to conventional antibiotics calls for alternative antimicrobial strategies. This study introduces a novel approach to acute wound healing by incorporating epitope-imprinted spongy cover materials with antimicrobial properties, using Tannic acid (TA) as the active agent within biocompatible cryogels imprinted with gallic acid. The spongy materials were synthesized and characterized through Fourier Transform Infrared Spectroscopy (FTIR), swelling tests, and Scanning Electron Microscopy (SEM) to assess their structural and physicochemical properties. The antimicrobial efficacy of the cryogels, loaded with 1.5, 3, 5 mg/mL of TA concentrations, was tested against *Staphylococcus aureus* and *Escherichia coli*, common pathogens in wound infections. The highest inhibition zone was determined to be 15 mm for *S. aureus* and 12 mm for *E. coli*. Maximum TA adsorption was 210.27 mg/g for eMIP and 24.74 mg/g for NIP. Cumulative release studies revealed the highest release rate occurred within the first 2 h. TA release kinetics indicated a non-Fickian diffusion mechanism. Additionally, the biocompatibility and potential cytotoxicity of the spongy materials, including TA-loaded variants, were assessed using the MTT assay on cultured cells. The results confirmed that the spongy materials are non-toxic and do not inhibit cell proliferation, supporting their suitability for acute wound healing. This study demonstrates that TA-loaded epitope-imprinted Poly(2-hydroxyethyl methacrylate) (pHEMA)-based spongy materials possess antimicrobial properties, making them potential candidates for wound and burn dressing applications.

**Graphical Abstract:**

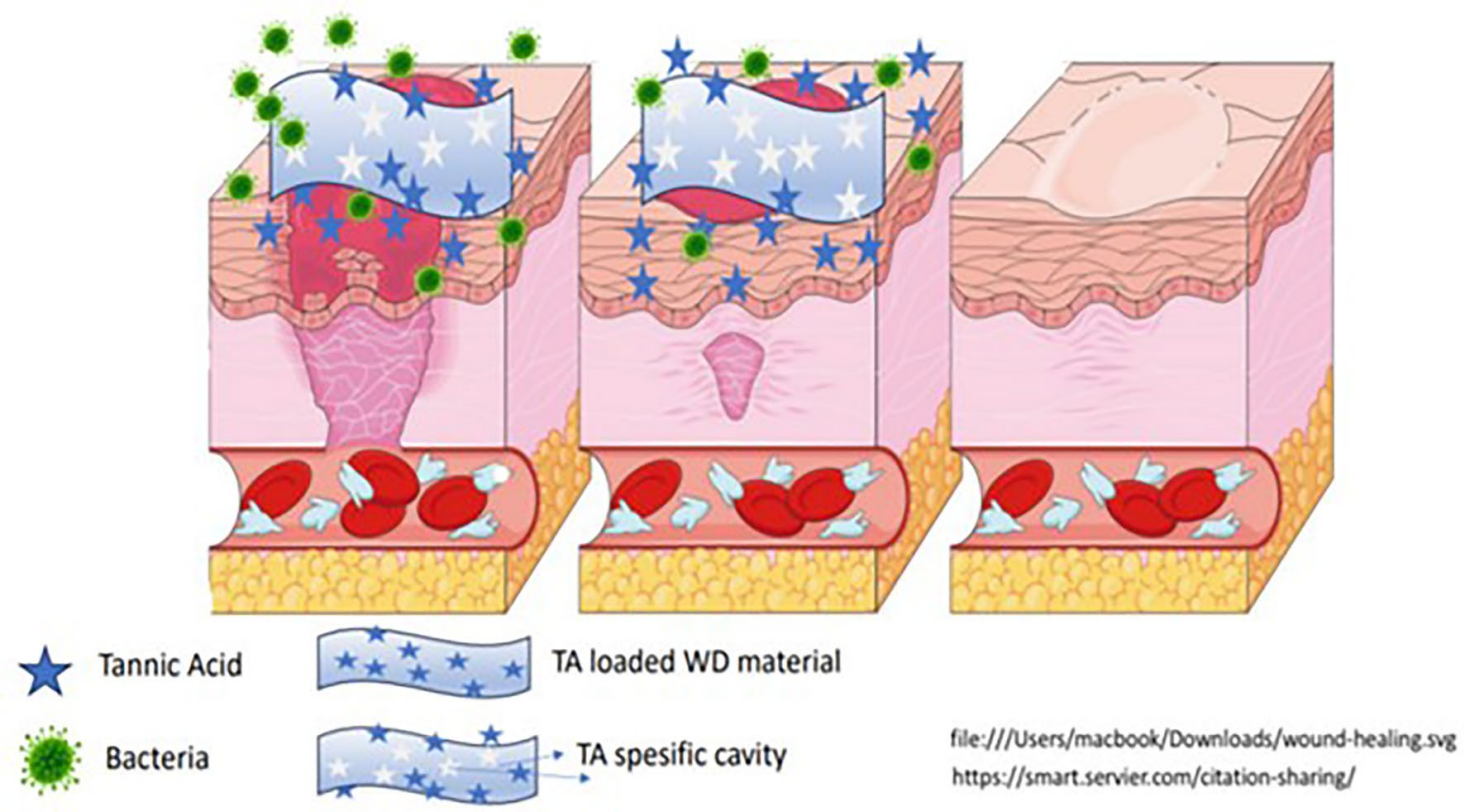

## Introduction

Tannic acid (TA) is a natural substance found in various plants and has antimutagenic, antioxidant, anticancer, antibacterial properties (Chaplin 1985; Kim et al. 2010; Ahmad 2014). Tannic acid is a complex polyphenol that is structurally composed of a central glucose unit to which ten gallic acid molecules are attached. The bioactive properties of TA allow it to neutralize free radicals, which are the cause of many diseases, such as diabetes, Parkinson’s, Alzheimer’s, allergies, and heart problems. The ability of TA to interact with macromolecules and biopolymers due to its hydroxyl groups makes it a desirable pharmacological candidate (Sagbas et al. 2015), gain interest as a polymer additive materials for biomedical purposes. As an antioxidant, TA is well known for its capacity to scavenge free radicals, defending cells from oxidative damage (Nakamura et al. 2003; Ahmad 2014). A range of harmful bacteria and viruses, including *Escherichia coli*,* Klebsiella pneumonia*,* Listeria monocytogenes*,* Staphylococcus aureus*,* Helicobacter pylori*, HIV and influenza are inhibited by TA’s antimicrobial action (Dai et al. 2014; Dabbaghi et al. 2019).

Because of their varied chemical structures, modes of action, and range of activity, antimicrobial compounds can be categorized in a wide variety of ways. Polymeric biocides, another name for antimicrobial polymers, are a class of materials that are intended to either kill or stop the growth of microorganisms. These polymers are designed to either naturally have antimicrobial qualities or to incorporate antimicrobial agents. The need for efficient antimicrobial materials in a variety of applications, such as food packaging, textiles, water treatment, and medical devices, to stop microbial contamination and biofilm formation, and to fight the growing problem of antimicrobial resistance, has fueled their development (Palza 2015; Gleckman 2001; McDonnell and Russell 2001; Lode 2009; Jain et al. 2014). Cryogels, antibacterial hydrogels, biopolymers (Khan et al. 2023a, b)., collagen-based materials (Wu et al. 2021)., metal-organic structures (Zhao et al. 2015) and polymeric materials are used to support soft tissue regeneration in infected wound healing (Khan et al. 2024; Prasathkumar and Sadhasivam 2021). These materials optimize wound healing with their infection control and biocompatibility properties. Ongoing research focuses on enhancing their efficacy, specificity, and biocompatibility, as well as addressing environmental concerns associated with their use.

Controlled release systems have garnered significant attention from researchers in the biomedical and pharmacological fields. The advancement of novel materials and technologies has led to the incorporation of these systems into therapeutic applications with the primary goal of enhancing drug safety and optimizing therapeutic efficacy (Langer 1993; Park 2014). In controlled release systems, it is aimed to release the molecules at the optimum value in a stable manner over a long period of time, without distinction of high or low dose (Huang et al. 2016). Molecular imprinting technology is widely used technology to develop drug relase systems recently. Molecularly imprinted polymers (MIPs) synthesized by interacting functional monomers with target molecule via secondary interactions ie.hydrogen bonding, electrostatic interactions or hydrophobic effects etc. Polymerization occur generally in presence of a cross-linker to provide structural integrity to the polymer. After polymerization, the template molecule removed from the polymeric structure, which leaves behind a cavity that is complementary in shape and functionality to the template molecules. Epitope imprinting is a specific area of molecular imprinting technology, focused on creating polymer matrices that can specifically recognize and bind to epitopes. An epitope, can be referred as a particular part of a complex molecule. The epitope imprinting approach, represents a significant advancement in molecular imprinting technology, specifically addressing the challenges of molecular complexity, flexibility, and the high costs associated with traditional imprinting methods (Singh et al. 2017). The key advantage of epitope-imprinted polymers is their high specificity and selectivity for the target epitope, which allows for the selective recognition and binding of specific parts in a complex molecule, making them valuable tools in biochemical and medicinal research. The epitope-imprinted polymers can be used in various applications, including biosensing, drug delivery, affinity separation and controlled release (Singh et al. 2017; Khumsap et al. 2021; Wang et al. 2021). In this study GA is used as an epitope of TA due to ten gallic acid molecules in its structure. Cryogels are three-dimensional gel matrices prepared with partially frozen monomeric or polymeric solutions. (Okay 2014). Cryogelation process happens polymerization in presence of ice crystals and the development of interconnecting pores following the dissolution of ice crystals in polymer network (Rogers and Bencherif 2019). Large pore diameters, short diffusion paths, strong biocompatibility, flexibility and great mechanical strength are just a few of the advantages of cryogels that have made them crucial components of bioseparation, purification and release applications (Kirsebom et al. 2010).

In this study, epitope-imprinted spongy cover materials were prepared to develop controlled tannic acid release antimicrobial spongy cover materials. pHEMA based gelatin included biocompatible spongy cover materials were prepared by imprinting gallic acid unit as an epitope of tannic acid. Tannic acid loaded spongy cover materials were then investigated for their use for effective controlled release antimicrobial wound healing biomaterial.

## Materials and methods

### Materials

Tannic Acid (TA), Gallic Acid (GA), 1- vinylimidazole (VIM), glutaraldehyde, 2- hydroxyethyl methacrylate (HEMA), Ethylene Glycol Dimethacrylate (EGDMA), Gelatine, ammonium persulfate (APS) were purchased from Sigma-Aldrich (St. Louis, MO, USA). N, N,N’,N’-tetramethylene diamine (TEMED) was supplied from BioRad. All other chemicals used in this study were supplied from Sigma-Aldrich (St. Louis, MO, USA). Required solutions were prepared using ultrapure Type 1 water (DI) with a conductivity of 18 MΩ-cm at room temperature and analytical grade reagents. Cultures of *S. aureus* (29213) and *E.coli* (25922) were obtained from American Type Culture Collection (ATCC).

## Synthesis of molecularly epitope imprinted and non-imprinted pHEMA based cryogels

GA-VIM pre-complex prepared using GA: VIM at a ratio of 1:8 (mole: mole), by dissolving GA (0.118 mmol) and VIM (0.944 mmol) in 1 mL of DI. Complexation completed at + 4 ℃ for 2 h. Cryogel synthesized as follows; 0.05 g of gelatin dissolved in 13.5 mL DI at 45 ℃, by stirring magnetically for 1 h. Then the pre-complex was added to the the gelatin solution as soon as cooled to room temperature. Then, HEMA (1.3 mL), EGDMA (0.506 mL) and glutaraldehyde (25 µL, 25%) were added into the final mixture and stir for 1 min. in an ice bath. 20 mg of APS and 25 µL TEMED were added to initiate cryogelation. The obtained polymer solution poured into the petri dish (90 mm X 17 mm) kept at − 18 ℃ for polymerization. After 24 h the cryogels thawed at room temperature to obtain interconnected 3D macropores and washed with DI water for several times to remove impurities followed by cutting in 18 mm (dm). Spongy cover materials were kept at + 4 ℃ in DI until use. The same steps were conducted for the synthesis of NIP, without addition of GA to provide control spongy cover materials. GA epitope template then removed from the synthesized spongy cover materials by interacting with 35 mL of PBS (10 mM, pH 7.4) containing 0.5 M NaCl, for 2 h using rotator. The removed GA amount determined spectrophotometrically at 273 nm, and the template removal studies were performed until GA was not detected (Zenger and Peşint 2022).

## Characterization studies

The swelling behavior of spongy cover materials was measured using 3 samples of each eMIP and NIP cryogels. The swollen weights, dry weights (lyophilized for 24 h at 0.001 mbar and **− 50 ℃**) and squeezed weights (immersed in an absorbent surface) of each sample were measured using a precision balance. The measurements for each sample were repeated 3 times.

The cryogels swelled in DI and recorded as Ws then the cover materials were lyophilized (Lyophilizer, BK-FD10P, Biobase, China), and the weights recorded as W_0_ and the swelling ratio (%) is calculated according to Equation given below:

1 $${\text{Swelling ratio }}\% {\text{ }} = {\text{ }}\left[ {\left( {{\text{W}}_{{\text{s}}} {-}{\text{ W}}_{0} } \right){\text{ }}/{\text{ W}}_{0} } \right]{\text{ }} \times {\text{ 1}}00\%$$

The total mass of the monomers used in cryogel formation was determined (m_T_). The polymerization yield (%) was calculated according to equation given below:

2 $${\text{Polymerization Yield }}\left( \% \right){\text{ }} = {\text{ }}\left( {{\text{m}}_{{{\text{dry}}}} /{\text{ m}}_{{\text{T}}} } \right){\text{ }} \times {\text{ 1}}00\%$$

The cryogels were swelled in DI water, weighed and recorded as m_swollen_, then swollen cryogels were squeezed to remove water from macropores, weighed and recorded as m_squeezed_. Then the percentage volume of supermacropores in cryogels was calculated using the following equation;

3 $${\text{Macroporosity }}\left( \% \right){\text{ }} = {\text{ }}({\text{m}}_{{{\text{swollen}}}} - {\text{m}}_{{{\text{squeezed}}}} )/{\text{m}}_{{{\text{swollen}}}} \times {\text{ 1}}00\%$$

eMIPs and NIPs ‘chemical structures were characterized using Fourier Transform Infrared (FTIR) spectrometer (Jasco, FT/IR-6700, USA). The synthesized eMIPs and NIPs were placed in the device in powder form for FTIR analysis. N_2_ gas was introduced into the sample chamber for 10 minutes to minimize interference from environmental gases and vapors on the sample spectrum. The spectrum was measured in the range of 4000 − 400 cm^−1^ wavenumbers.

SEM analysis was used to investigate the morphological structures of eMIP and NIP cryogels (SEM, JEOL JSM 5600, Jeol Co., Tokyo, Japan). Samples were lyophilized before SEM analysis. After drying, the cryogel cover materials samples fixed onto the SEM sample plate using a then SEM images of the samples were taken at different magnifications.

## Adsorption studies

The adsorption of Tannic Acid (TA) by Molecularly Imprinted Polymers (eMIP) and Non-Imprinted Polymers (NIP) was examined in a batch system at 25 **℃**, with a pH of 7.4. Initially, equal amounts of eMIP and NIPs were weighed and placed into separate falcon tubes. To investigate the effects of equilibrium TA concentration on the adsorption capacity, 5 mL of TA solutions with varying concentrations ranging from 1.5 to 10 mg/mL were added to the cryogel cover materials in the falcon tubes. These mixtures were then rotated at 20 rpm at 25 **℃** for 2 h to achieve interaction. The amount of TA adsorbed was determined using a UV-VIS spectrophotometer at 277 nm and was calculated as follows:

4 $${\text{Q }} = {\text{ }}\left( {{\text{C}}_{{\text{o}}} - {\text{C}}_{{\text{f}}} } \right){\text{ }}\times{\text{ }}\left( {{\text{V}}/{\text{m}}} \right)$$

here, Q is TA adsorption capacity (mg/g), C_o_ and C_f_ is TA concentrations before and after interaction with cover materials (mg/mL), V is volume of the TA solutions (mL), m is the dried mass of the cover materials (g).

## Release analyses

TA release studies were achived by measuring released TA amount from cover materials for 24 h in a rotator that operates at 20 rpm and at a constant room temperature of 25 **℃**. The release amount TA from cryogel cover materials were examined using cryogel cover materials loaded with different TA concentrations (1.5, 3, 5, 7 and 10 mg/mL) separately in 10 mL DI. TA samples were taken at specific time intervals and fresh DI was added to the medium to maintain constant driving force, in each step. The released TA amount was determined spectrophotometrically at 277 nm, and the cumulative release percentages of the samples were determined according to following equation,

5 $$Cumulative~Release~Rate~\left( \% \right) = \left( {\frac{{The~amount~of~TA,~\frac{{~mg}}{{mL}}}}{{The~maximum~amount~of~TA~,\frac{{mg}}{{mL}}~}}} \right) \times 100$$

here, the cumulative release rate of TA (%), expresses the percentage of cumulative release amount of TA found in samples taken at different times. The amount of TA in the sample refers to the TA concentration (mg/mL) in each sample. The maximum amount of TA in the sample refers to the maximum amount of TA that should be released from cryogel sample (Manjula et al. 2021; Varlık et al. 2023).

Mathematical release kinetic models have been developed for different conditions and materials to investigate the release processes of a compound from polymeric materials (Peppas et al. 2000; Çetin and Denizli 2015; Varlık et al. 2023). The Korsmeyer-Peppas model is one of the commonly used modelling methods to characterize drug release. This model assumes that the release rate is completely dependent on the physical properties of the molecular formulation of loaded molecule and its diffusion rate.

The Korsmeyer-Peppas kinetic release model is expressed as follows:

6 $${\text{M}}_{{\text{t}}} /{\text{M}}_{\infty } = {\text{k}}\times{\text{t}}^{{\text{n}}}$$

here Mt is amount of drug released at time t, M_∞_ is the total amount of drug released at infinite time, k is a constant incorporating structural and geometric characteristics of the dosage form, t is the release time, and n is the release exponent that characterizes the drug release mechanism. This model is widely used to analyze and predict drug release behavior from various pharmaceutical formulations. Equation 6 can be linearized as following equation:

7 $${\text{log }}(M_{\infty } /M_{t} ~){\text{ }} = {\text{ log}}\left( k \right) + n\times{\text{log}}\left( t \right)$$

The exponent n is determined by plotting the data from Eq. 7 on a logarithmic scale and calculating the slope using linear regression. The value of k corresponds to the intercept of this line. The value of n can be interpreted as follows (Varlık et al. 2023):

Fick’s laws of diffusion describe the way that particles, like atoms, ions, or molecules, will move with respect to concentration gradients (Çetin and Denizli 2015; Won and Ramkrishna 2019). Whereas the first law deals with the flux that is derived by the gradient, the second one describes the evolution of the concentration due to diffusion with time (Won and Ramkrishna 2019). These laws are essential for the prediction and analysis of various sorts of diffusion processes in many scientific disciplines. Uniform diffusion controlled by Fick’s diffusion law defines as if *n* = 0.5; a situation where a different diffusion mechanism (e.g. polymer dissolution) is in effect define if 0.5 < *n* < 1; if *n* = 1 describes Pure Fick diffusion control and if *n* > 1 indicates situations where drug release is controlled by another mechanism (for example, radial diffusion). Fickian diffusion control is typically observed in homogeneous and isotropic environments. In such cases, material transfer occurs regularly and linearly according to the concentration gradient. For instance, the diffusion of gases or small molecules is often well-described by Fick’s law of diffusion (Çetin and Denizli 2015). However, Fickian diffusion control is not always applicable, especially in complex and heterogeneous systems (such as polymer materials, gel structures) or in cases of non-Fickian Diffusion. In these situations, diffusion may need to conform to a more complex model, such as the Korsmeyer-Peppas model, rather than following Fickian principles (Peppas et al. 2000; Varlık et al. 2023).

### Antimicrobial studies

Antibacterial properties of the synthesized eMIP and NIP cryogels were examined. Mueller-Hinton Agar (MHA) solid medium was prepared for antibacterial studies. *S. aureus* and *E. coli* strains were used to examine the effect of eMIP and NIP cover materials on gram-positive and gram-negative bacteria, respectively. The McFarland standard was used to prepare bacterial suspensions. McFarland is a standard used as a turbidity standard in the preparation of microorganism suspensions. The 0.5 McFarland standard was used in this study. This standard is frequently applied during the preparation of bacterial inoculums, especially for performing antimicrobial susceptibility tests.

*E. coli* and *S. aureus* were prepared in PBS (pH:7.4), and their turbidity was adjusted by comparing them with McFarland 0.5 solution. After the physical evaluation was completed, the samples were checked by taking measurements at 600 nm using a UV-VIS spectrometer, and thus 10^8^ cfu/mL bacterial inoculums were prepared. Then, 1 mL inoculum from each microorganism (*E.coli* and *S.aureus*) was taken and spread onto the MHA medium prepared in each petri dish using an L-shaped rod.

Antimicrobial analysis were carried out according to disc diffusion method and the experiments were carried out in triplicate. eMIPs loaded with 1.5, 3, and 5 mg/mL TA were placed in the middle of MHA medium containing microorganisms. The same procedure was performed with NIPs as the control group. It was incubated at 35 ± 0.1 **℃** for 24 h. According to the zones of inhibition (mm) results obtained, the antibacterial effectiveness of eMIP and NIP groups on *E. coli* and *S. aureus* strains was calculated.

## In Vitro Cell studies

The MTT assay can be utilized to assess biocompatibility, offering insights into how biomaterials interact with cellular systems. MTT test was used in this study to investigate biocompatibility property by the cytotoxic effects of eMIP and NIP cover materials on cells. The MTT (3-(4,5-dimethylthiazol-2-yl)−2,5-diphenyltetrazolium bromide) assay is a widely used method to assess cell viability and proliferation in cytotoxicity studies. It works by measuring the conversion of MTT into purple formazan crystals by metabolically active cells. The intensity of the resulting color is directly proportional to the number of viable cells. Typically, higher absorbance values indicate greater cell viability, while lower values suggest cytotoxic effects of tested compounds. By comparing absorbance values of treated samples to controls, the relative cytotoxicity of compounds can be determined. In the investigation of eMIP and NIP cover materials’ cytotoxic effects on cells, the MTT assay provides crucial insights into their potential biomedical applications.

Sterilization and preparation of materials for cell culture studies were achieved, briefly; the eMIP and NIP were cut to 10 mm diameter by perforator and kept in 70% ethanol for 1 h for sterilization then eMIP and NIPs were washed with sterile distilled water twice. 0.005 M Tannic acid solution was filter sterilized. The sterilized materials were kept in sterile tannic acid solution for 2 h for adsorption. After adsorption took place, the materials were transferred to a 24-well cell plate.

In vitro cytotoxicity test was performed with HaCaT (CLS 300493, DKFZ, Heidelberg, Germany) cell line. 10 × 10^4^ cells were seeded on both eMIP and NIP materials. A control group was also created by seeding 10 × 10^4^ cells into a well plate without any material. Cells were cultured with DMEM-F12 (Dulbecco′s modified Eagle′s medium/Nutrient Mixture F-12) (Gibco, Thermo Fisher) supplemented by 10% Fetal Bovine Serum (Gibco, Thermo Fisher) in a humidified incubator at 37 **℃** with 5% CO_2_ for 24 and 48 h.

Then the MTT Cell Viability Assay were achieved. Cytotoxicity was investigated by MTT cell viability assay. After incubation periods of 24 and 48 h, the cell medium was removed, and cells were washed twice with PBS. 0.5 mg/mL MTT solution was given to the cells and incubated. When formazan crystals became visible, approximately 2 h, the MTT solution was aspirated. DMSO was added to dissolve formazan crystals and left in the shaker for 30 min. After the formazan crystals were completely dissolved, the absorbances were read at 570 nm. All groups were tested as triplicate. The cell viability percentage was calculated considering the control group as 100. The following formula was utilized for the calculation.

8 $${\text{Cell viability }}\left( \% \right){\text{ }} = {\text{ }}\left( {{\text{Average of test cells }}/{\text{ Average of control cells}}} \right){\text{ }} \times {\text{1}}00$$

In the given equation, the term “test cells” refers to the assessment of cell viability for cells cultured on materials, whereas the term “control cells” pertains to the evaluation of cell viability for cells cultured on a standard culture plate.

## Results

### Synthesis of molecularly epitope imprinted and non-imprinted pHEMA-gelatin cryogels

**Fig. 1:**
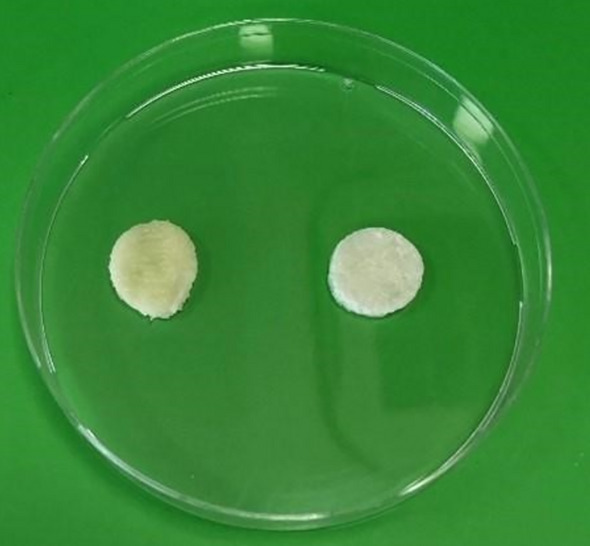
Optical photographs of eMIP (left) and NIP (right) cover materials

Synthesized cryogels, eMIP, which contains GA was observed in darker yellow tones, while NIP, which is the control group, was observed in light yellow tones and a color closer to white (Fig. 1).

**Fig. 2:**
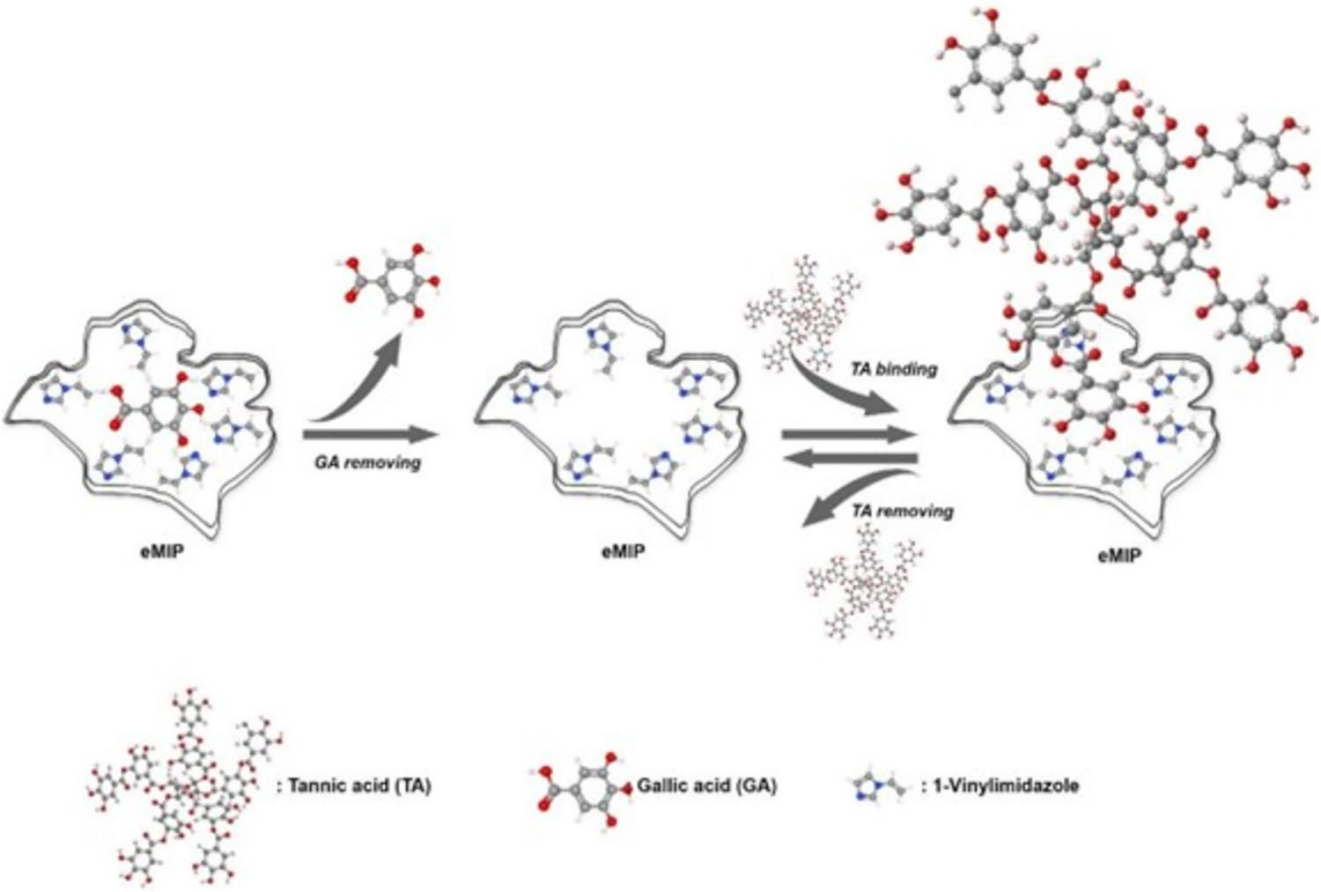
Illustration of the process of GA-epitope imprinting on eMIP and its removal from the structure. Reprinted with permission from (American Chemical Society, 2018).

GA was used as the epitope of TA. Before loading TA to the eMIPs the GA should be removed from the cover materials properly to obtain GA unit spesific recognition cavities. GA spesific cavities obtained, by applying an elution agent (0.5 M NaCl in PBS pH 7.4) to the eMIP. After four cycles of elution, approximately 96% of GA unit molecules were removed from the cavities. The obtained GA spesific cavities then used for the selective binding of TA (Fig. 2).

### Characterization studies

Swollen samples (18 mm in diameter) were used to conduct swelling test studies. Swelling rate, porosity and polymerization yield were determined by swelling tests. It was observed that the inclusion of GA in the structure reduced the gelation efficiency of the cryogel, but it did not have a negative effect on the macroporosity and swelling ratios (Table 1).

**Table 1:** Polymerization yield, swelling rate and porosity ratios of eMIP and NIP

Polymers	Polymerization yield (%)	Swelling rate (%)	Porosity (%)
NIP	87	95	80
eMIP	85	98	83

**Fig. 3:**
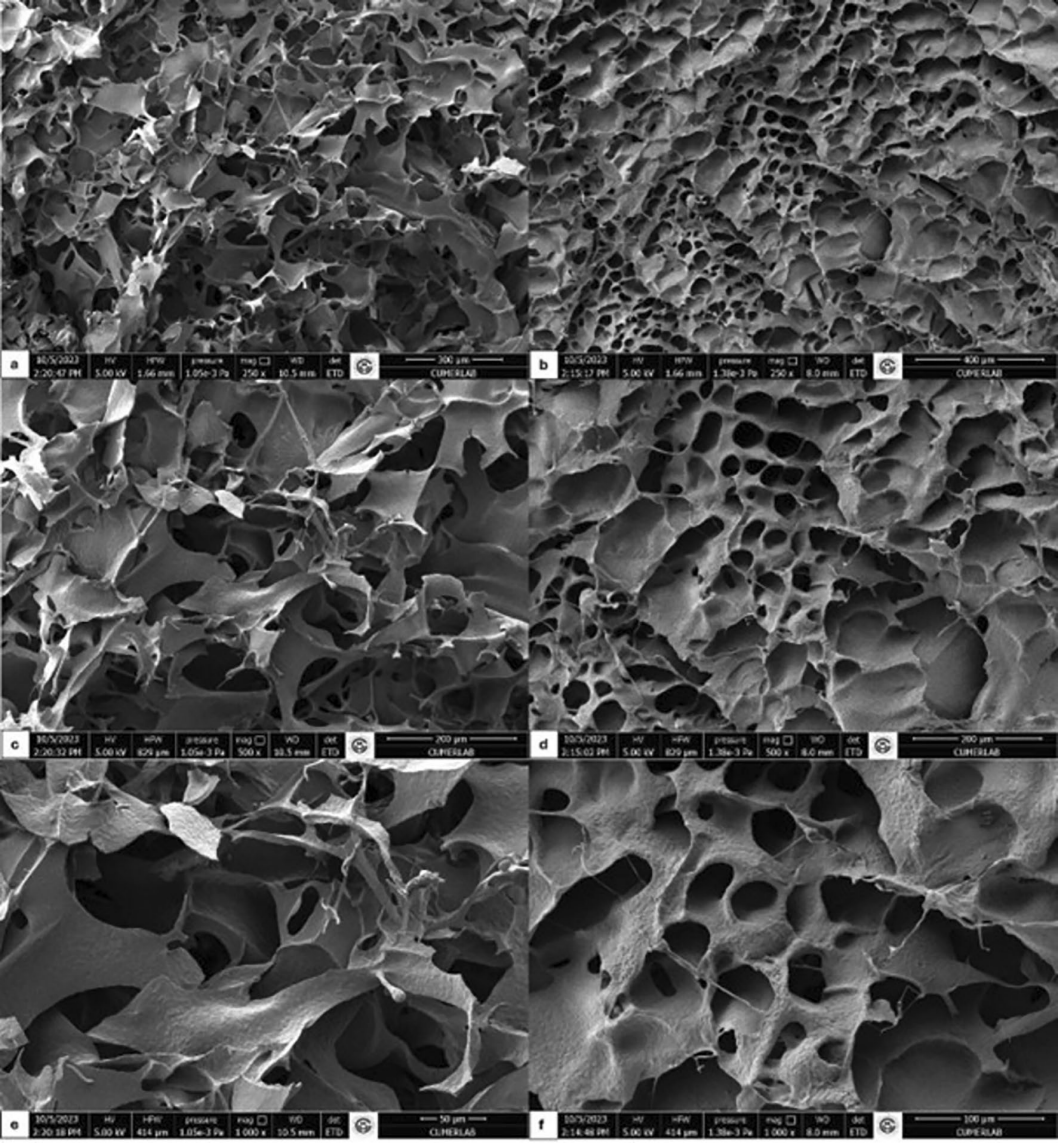
SEM images of NIP (**a**, **c**, **e**) and eMIP (**b**, **d**, **f**) (250×, 500×, 1000×, respectively)

SEM images of eMIP and NIP showed that both of them have homogeneously distributed interconnected macropores (Zenger and Peşint 2022) (Fig. 3).

**Fig. 4:**
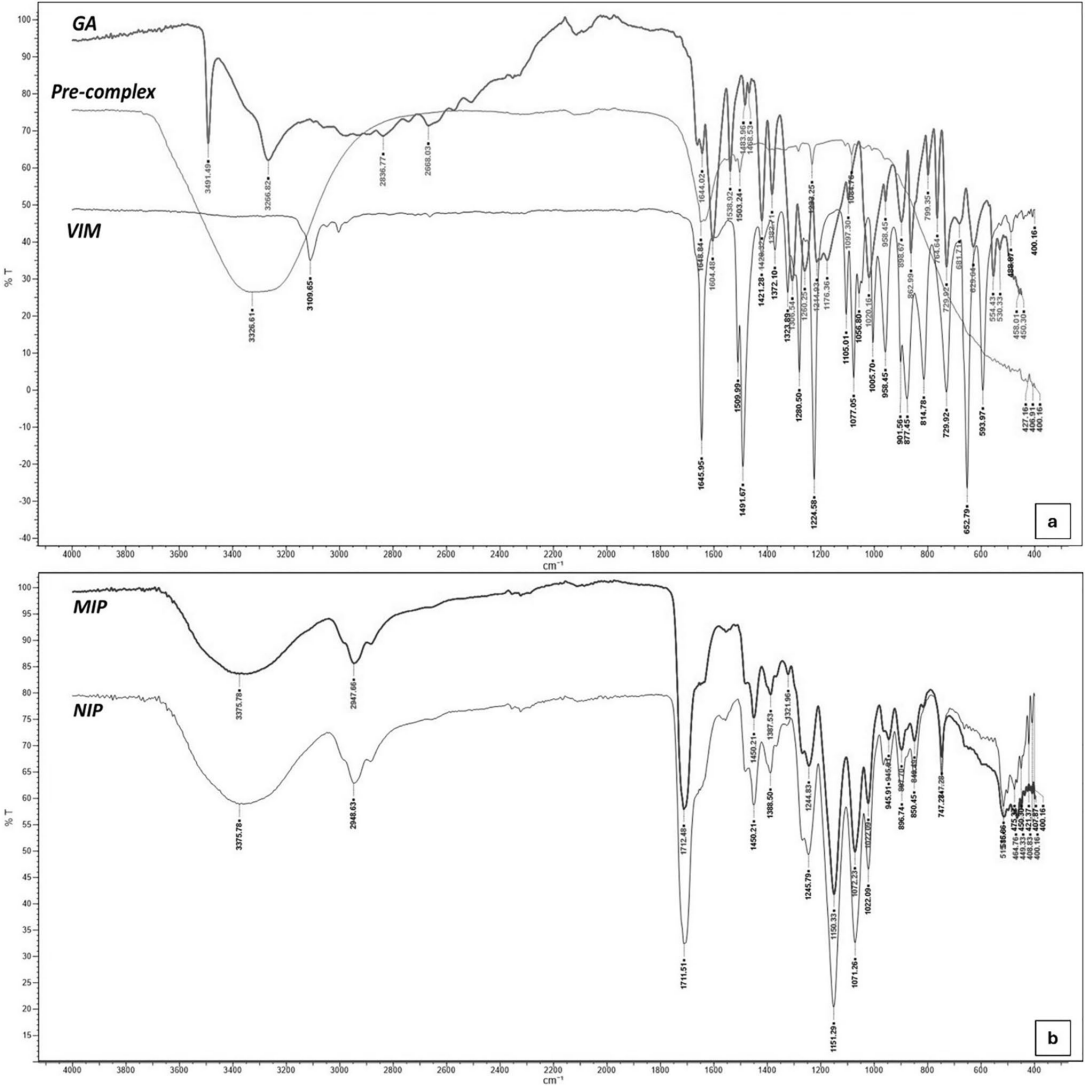
FTIR Spectrum of **a** GA, VIM and GA: VIM (1:8 /n: n) pre-complex, **b** eMIP and NIP

Structural analysis of cryogels was evaluated via FTIR-ATR for eMIP and NIP. The spectra of GA, VIM and GA-VIM precomplex (1:8) are given below, respectively (Fig. 4a).

When the FTIR spectrum of GA is examined, the intense peak at 3491 cm⁻¹ is indicative of a robust O–H stretching vibration, pointing towards the presence of hydroxyl groups in the GA molecule. At 3266 cm⁻¹, a strong O–H stretching peak emerges, specifically attributed to the carboxylic acid group. This observation reinforces the identification of the carboxylic acid functional group within the GA. The region between 2600 and 2800 cm⁻¹ displays medium-intensity peaks corresponding to C–H stretching vibrations. These peaks affirm the presence of carbon-hydrogen bonds in the compound, contributing to the overall structure of GA. The peaks at 1539 and 1604 cm⁻¹ are associated with C=C stretching vibrations, characteristic of aromatic rings. These peaks serve as a distinct marker for the presence of aromatic rings within the molecular structure of GA. In the range of 1000–1300 cm⁻¹, strong peaks are observed, indicating C–O stretching vibrations. These peaks are particularly relevant to the functional groups involving oxygen atoms and contribute essential information regarding the molecular configuration of GA. Finally, the strong peaks within the 700–900 cm⁻¹ range are attributed to C–H bending vibrations. These peaks emphasize the presence of carbon-hydrogen bonds and their specific bending motions within the GA molecule. In the spectrum of VIM, The peak at 3109 cm⁻¹ signifies the C-H stretching of the aromatic ring, confirming the presence of the structural feature of an aromatic ring within the molecule. Medium intensity peaks around 1646 cm⁻¹ predominantly represent the C=C double bonds and N–H bending associated with the aromatic ring. This indicates the distinct impact of double bonds and amine groups on the FTIR spectrum. The peaks at 1509 and 1492 cm⁻¹ correspond to C–N stretching, demonstrating the presence of C–N bonds within the molecule. These peaks specifically highlight the stretching vibrations of these bonds. The peaks in the range of 1300–1400 cm⁻¹ indicate strong C-N stretching vibrations within the aromatic ring. These peaks are unique to VIM and leave a prominent signature in the FTIR spectrum. When the FTIR spectrum of GA-VIM pre-complex is examined; The large peak observed at 3326 cm⁻¹ is attributed to O–H stretching vibrations, believed to be associated with GA. This broadening suggests hydrogen bonding, emphasizing the interaction between GA-VIM. The peak at 1648 cm⁻¹ signifies C=C stretching vibrations and N–H bending, characteristics associated with aromatic rings. This observation supports the successful inclusion of the aromatic structures from both GA and VIM in the complex. The peaks at 1503 and 1233 cm⁻¹ are thought to be associated with VIM, specifically corresponding to C–N stretching vibrations within aromatic rings. This provides further evidence of the incorporation of VIM into the complex, emphasizing the interaction between the two molecules. Additionally, the peak at 1084 cm⁻¹ is attributed to C–H stretching vibrations, indicating the presence of carbon-hydrogen bonds. This observation further supports the successful formation of the preliminary complex. In conclusion, the FTIR analysis underscores the successful complexation of GA and VIM, as evidenced by the distinctive peaks associated with the individual components. The observed vibrational modes and peak patterns strongly indicate the formation of hydrogen bonds and interactions between the aromatic structures.

Below are the characteristic FTIR spectra for eMIP and NIP (Fig. 4b). Since the structures of eMIP and NIP are quite similar, their spectra also exhibit significant similarities. Despite the masking effect of the abundant content of Gelatin, HEMA and EGDMA in the structure, the presence of VIM and GA molecules has been successfully demonstrated. First of all, the wide O–H stretching band at 3376 cm⁻¹ seen in both spectra is the hydrogen containing gelatin and HEMA. The C-H stretching peaks at 2948 cm⁻¹ originate from the methylene groups of EGDMA and the methylene and methenyl groups of HEMA. The C=O stretching peaks at 1711 cm⁻¹ represent the carboxyl or ester groups of ethylene glycol dimethacrylate and HEMA. Additionally, the C–H deformation peaks at 1450 cm⁻¹ indicate the presence of methylene or methenyl groups, while the peaks at 1388 cm⁻¹ and 1245 cm⁻¹ reflect C–H deformations along with C–N or C–O stretching vibrations. Unlike in NIP, the band observed in eMIP at 1322 cm⁻¹ corresponds to amine group stretching bands, suggesting the presence of amine groups within the imprinted cavities. This observation may serve as evidence that eMIP forms more hydrogen bonds than NIP. The existence of this peak can also be attributed to the inclusion of the VIM molecule in the structure. However, the frequency shift at 1711 cm⁻¹, caused by C=O vibrations, to a higher frequency in eMIP, along with the difference in peak intensity compared to NIP, supports the presence of the pre-complex molecule in the structure. These variations observed in the spectra strongly indicate the incorporation of the GA-VIM pre-complex into the structure of eMIP.

### Adsorption studies

**Fig. 5:**
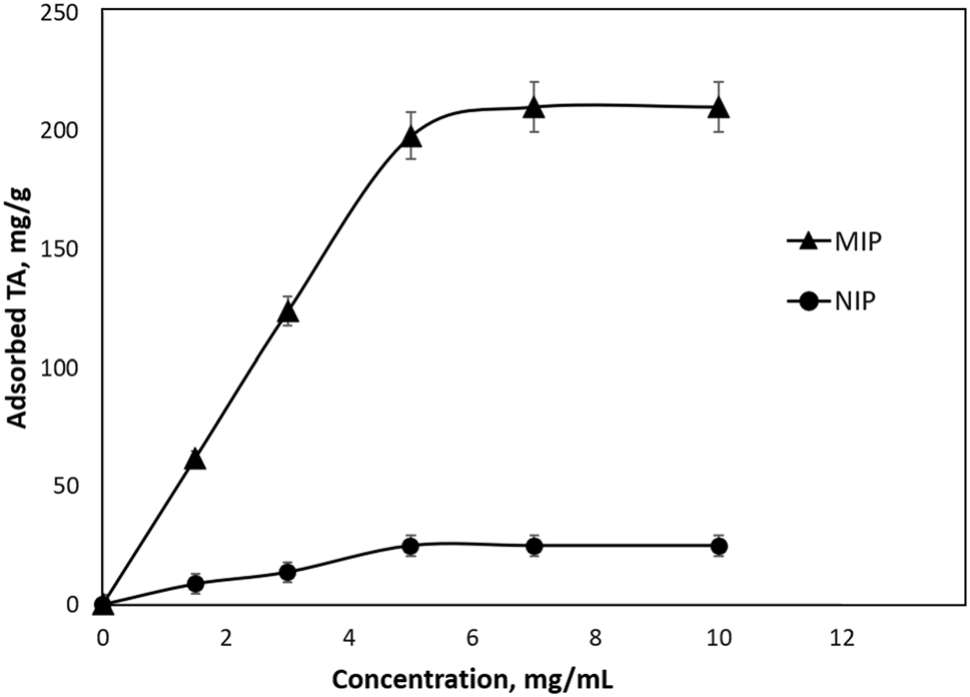
TA loading amounts of eMIP and NIP. m_dry_: 0.1155 g, V: 5 mL, time: 120 min, T: 25 ℃, pH: 7.4

GA epitope removed cavities were loaded with TA and the maximum TA loading capacity determined by interacting different concentrations of TA solutions (between 1.5 and 10 mg/mL) to the cover materials. The maximum TA adsorption for eMIP and NIP polymers was calculated as 210.27 mg and 24.74 mg per gram cover material, respectively (Fig. 5).

**Fig. 6:**
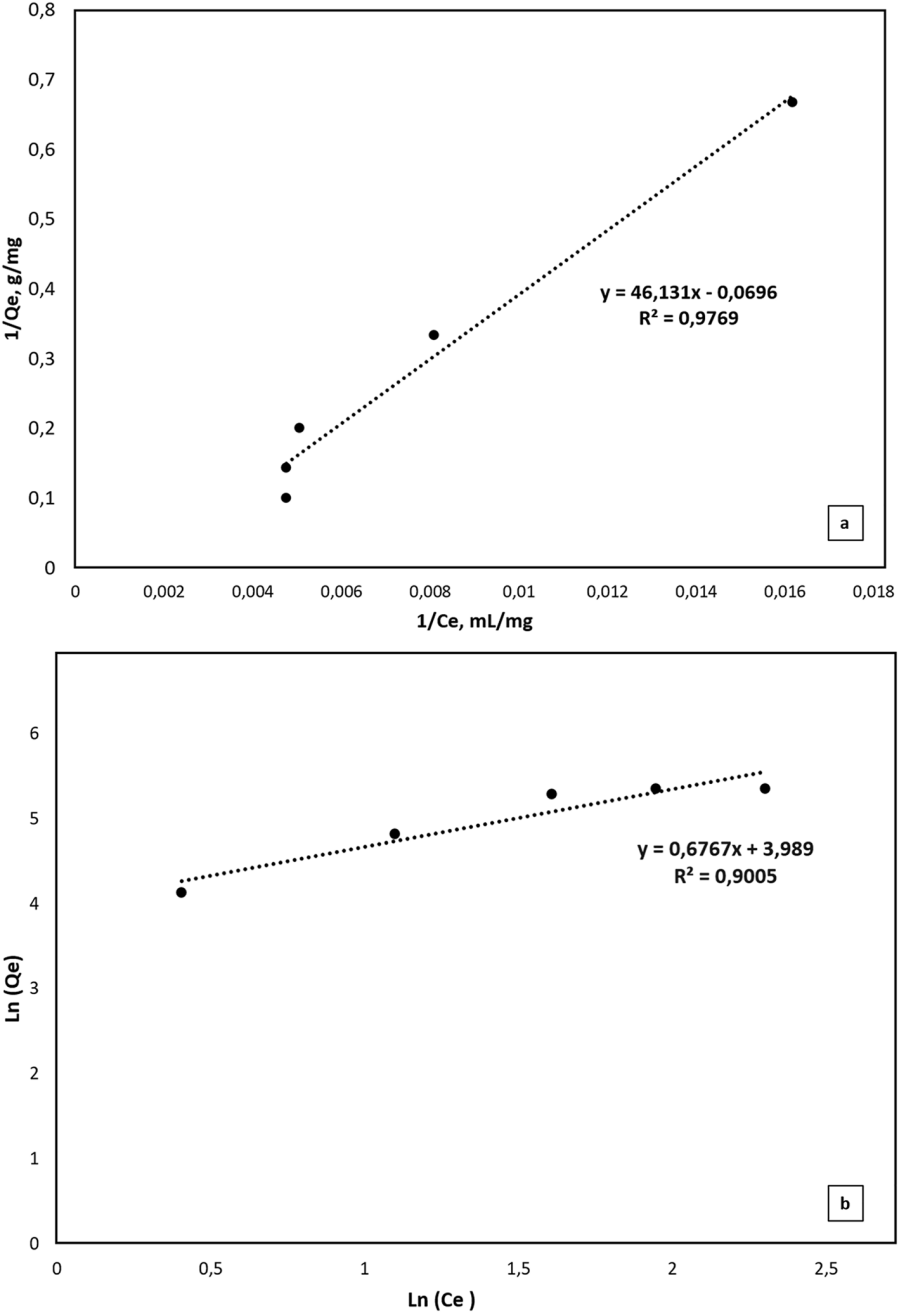
Adsorption isotherm plots of eMIP **a** Langmuir, **b** Freundlich

The Langmuir and Freundlich adsorption isotherm models were used to investigate the interaction behavior of stnthesized cover materials with TA. Figure 6 shows the Langmuir and Freundlich adsorption isotherms of TA adsorption on eMIP. The Langmuir adsorption isotherm describes monolayer adsorption in which it is assumed that all binding sites are homogeneous, energitally equal and adsorbed species don’t interact with each other within themselves (Foo and Hameed 2010). The Langmuir model is defined by the following equation:

9 $${\text{C}}_{{\text{e}}} /{\text{Q}}_{{\text{e}}} = {\text{ 1}}/{\text{Q}}_{{{\text{max}}}} + {\text{ 1}}/{\text{Q}}_{{{\text{max}}}} \times {\text{b}} \times {\text{C}}_{{\text{e}}}$$

When the Eq. 9 is linearized, the following equation is obtained:

10 $${\text{C}}_{{\text{e}}} /{\text{Q}}_{{\text{e}}} = {\text{ 1 }}/{\text{ }}\left( {{\text{Q}}_{{{\text{max}}}} .{\text{b}}} \right){\text{ }} + {\text{ }}\left( {{\text{C}}_{{\text{e}}} /{\text{Q}}_{{{\text{max}}}} } \right)$$

The C_e_ versus C_e_/Q_e_ plotted to obtain the intersection of the line formed gives 1/Q_max_*b, and its slope gives 1/Q_max_. Here Q_e_ is TA binding capacity (mg/g), C_e is_ equilibrium TA concentration (mg/mL), b is Langmuir constant (mL/mg), Q_max_ is maximum TA adsorption capacity (mg/g).

The maximum TA adsorption capacity (Q_max_) 588.23 mg/g, and the Langmuir constant (b) calculated as 0.08 mL/mg from the slope and cut of the graph **in** Fig. 6**(**Table 2**).**

The Freundlich isotherm describes multilayer adsroption, it assumes that the binding sites are not homogeneous, energitacaly not equal, adsorbed species can interact with each other within themselves. The equation for the Freundlich adsorption isotherm is as follows:

11 $${\text{Q}}_{{\text{e}}} = {\text{ Q}}_{{\text{f}}} \times {\text{C}}_{{\text{e}}} ^{{{\text{1}}/{\text{n}}}}$$

When the Eq. 11 is linearized, the following equation is obtained:

12 $${\text{lnQ}}_{{\text{e}}} = {\text{ lnQ}}_{{\text{f}}} + {\text{ 1}}/{\text{n}} \times {\text{lnC}}_{{\text{e}}}$$

here Q_e_ is TA binding capacity (mg/g); C_e_ is equilibrium TA concentration (mg/mL); Q_f_ is the Freundlich adsorption capacity of the adsorbent (mg/g); 1/n is the freundlich constant. The lnC_e_ against lnQ_e_ plotted to obtain 1/n and lnQ_f_ values from the slope and the intersect of the lines. Figure 6.b shows the Freundlich adsorption isotherm of TA adsorption of eMIP. The 1/n was found 0.6767 and the Q_f_ was calculated as 53 mg/g **(**Table 2**)**.

**Table 2:** Langmuir and Freundlich adsorption isotherm constants and related adsorption capacities of eMIP

Experimental	Langmuir Constants			Freundlich Constants		
Q_e_	Q_max_	b (mL/mg)	R^2^	Q_F_	n	R^2^
210.3	588	0.08	0.977	53.00	1.48	0.9

### Release analyses

**Fig. 7:**
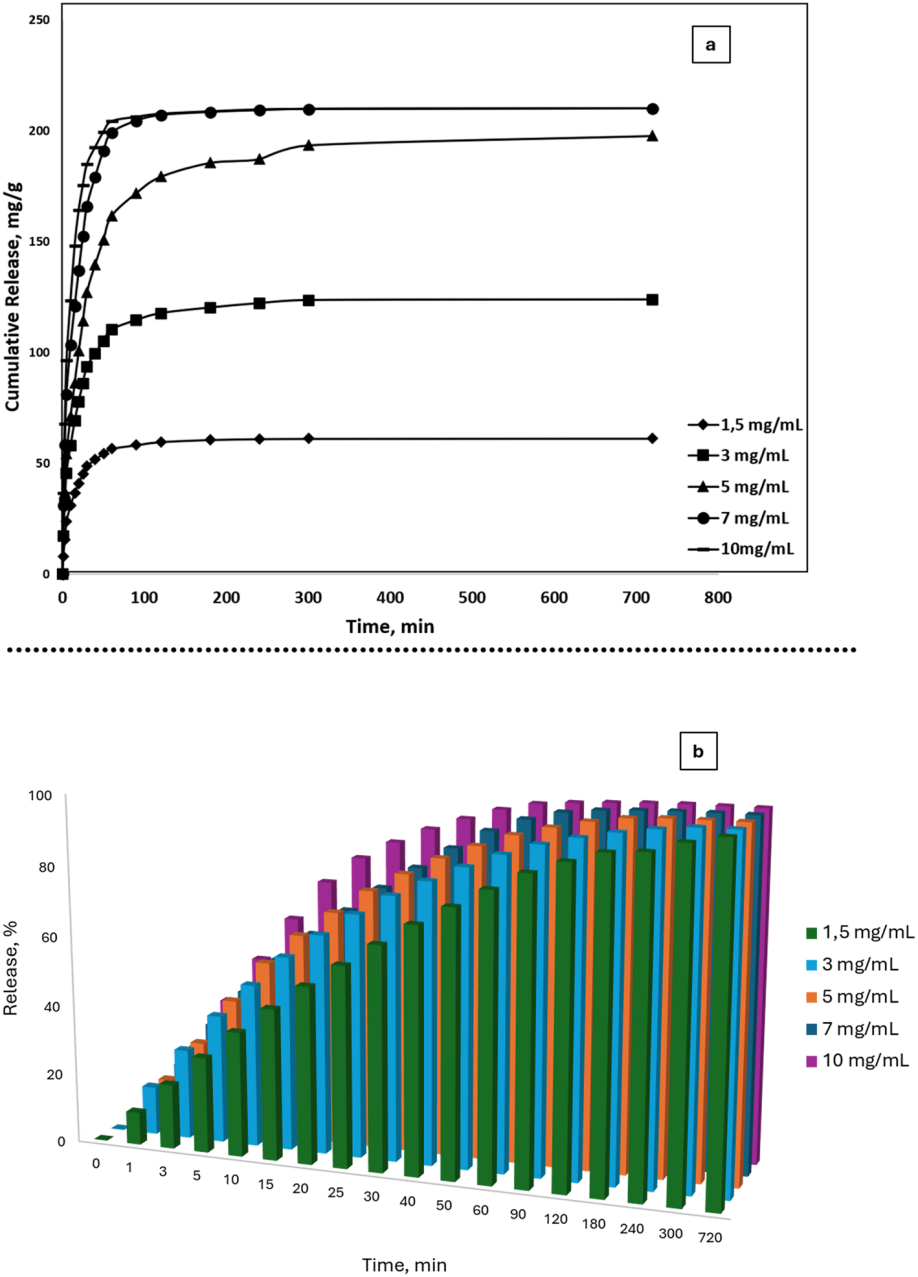
Effect of TA concentration on release depending on time (T: 25 °C, pH:7.4, DI) **a** Cumulative release (mg/g), **b** Percentage release (%)

Among the important parameters affecting the drug release rate is the amount of drug initially loaded into the polymeric system. In this study, where TA release from cryogel cover materials was examined, TA was loaded by adsorption at 5 different concentrations (1.5 mg/mL, 3 mg/mL, 5 mg/mL, 7 mg/mL, 10 mg/mL). In order to determine the effect of loading TA amount on release kinetics; release pH (7.4) and temperature (25 **℃**) were kept constant. Figure 7 shows that the release rate increases as the initial TA loading amount increases. Cumulative release analysis studies of TA loaded cryogels showed that the maximum release rate is reached in the first 2 h (Fig. 7a-b).

**Fig. 8:**
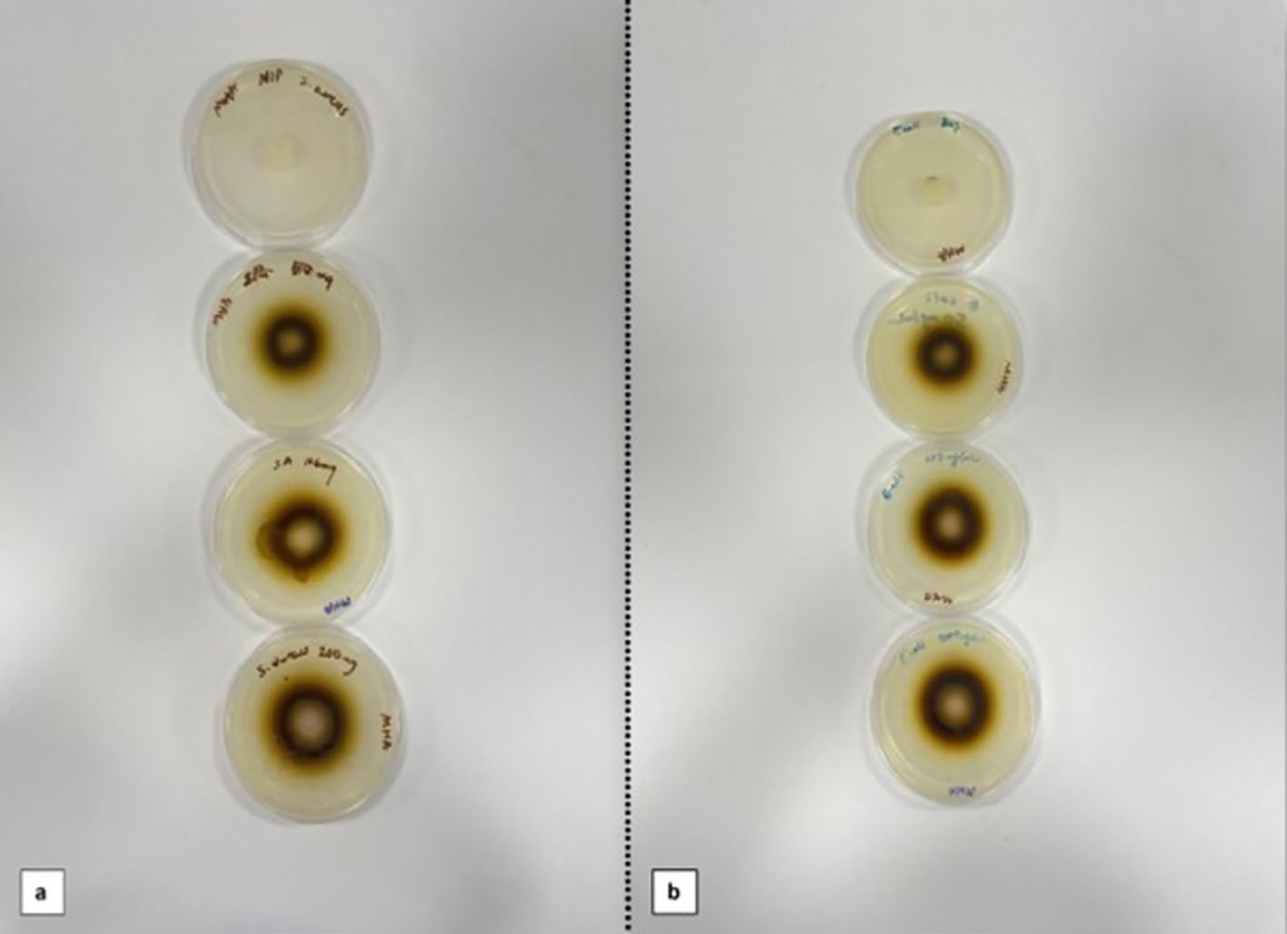
Optical images of antimicrobial eMIPs and NIPs against; **a** *S. aureus* and **b** *E. coli*

Time-dependent cumulative release percentage (%) rates of each cryogel evaluated in Fig. 7b. Cryogels released almost all of the TA in their structures nearly in 2 h. All release behavior almost the same for each cryogel cover materials (Fig. 8).

Time-dependent cumulative release rates of cryogels were evaluated with the parameters of Korsmeyer-Peppas kinetic release models (Table 3). The relative effect of macromolecular structure on the drug release mechanism can be easily determined by modifying the experimental data to Eq. 6. However, this equation is applicable only for the initial 60% of the total released drug.

Equation 6 defines the type of diffusion process that depends on the value of n and describes the overall transport behavior of the solvent in the polymer. Plotting the data from this equation on a logarithmic scale and using linear regression to calculate the slope allowed for the determination of the diffusional exponent, or n.through a diffusion process of a non-Fickian type.

Table 3 shows the release coefficients ​​n and k of eMIP cryogel cover materials containing different amounts of TA, and also the regression coefficients. As given in Table 3, n values are smaller than 0.5.

**Table 3:** N, k and R^2^ values obtained when the Korsmeyer-Peppas release kinetics model is applied

Initial TA concentration (mg/mL)	*n*	k	*R* ^2^
1.5	0.39	1.7	0.91
3	0.38	1.7	0.93
5	0.45	2.1	0.96
7	0.37	1.64	0.93
10	0.33	1.4	0.88

### Antimicrobial studies

eMIP cryogel cover materials loaded with three different concentrations of TA showed antimicrobial activity against *S. aureus* and *E. coli*. Inhibition zone diameters of cover materials were determined to be larger at highest concentration of TA which is 5 mg/mL according to 1.5 and 3 mg/mL. The highest antimicrobial activity with 15 mm inhibition zone was observed against *S. aureus*. On the other hand, inhibition zone of 12 mm was observed against *E. coli*.

**Fig. 9:**
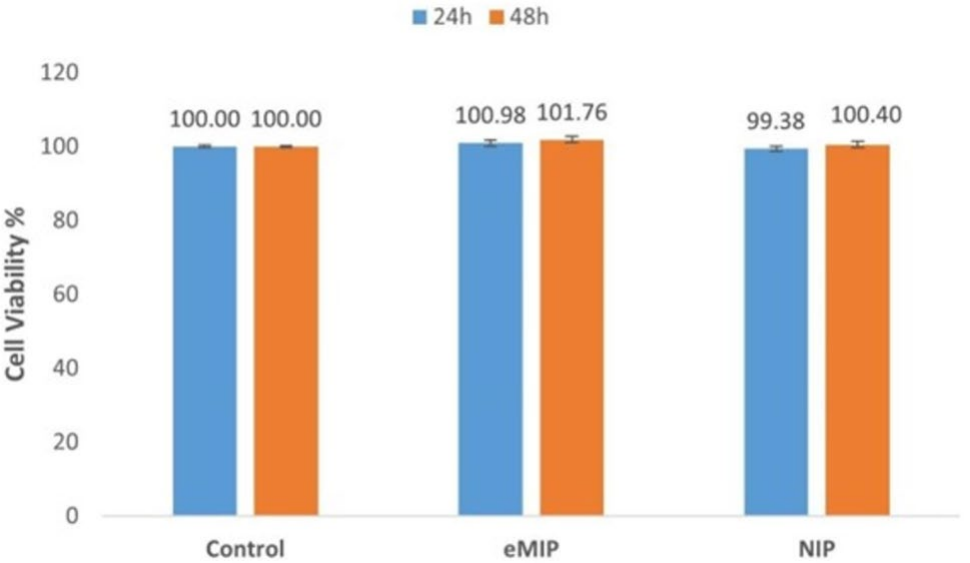
The viability of HaCaT cells cultured on synthesized cryogels

### In Vitro Cell studies

MTT assay results revealed that all synthesized materials had no cytotoxic effect on HaCaT cells and were suitable for cell growth (Fig. 9). It was determined that as the incubation time increased, cell proliferation in eMIP cryogels increased slightly and viability was slightly higher than in the 2D control group (100.98% and 101.76% respectively). Cell viability for the NIP cryogel was very close to the 2D control at both incubation times (99.38% and 100.4% for 24 and 48 h respectively). Indeed, cell viability for both materials showed no significant difference compared to the 2D control in either incubation period. This indicated that neither the material nor the TA loaded into it has a toxic effect on the cells and does not inhibit their proliferation.

## Discussion

In this study, eMIP and control group NIP spongy cover materials were successfully synthesized. Only when a qualitative inference is made, the darker, yellow tones observed in eMIP compared to NIP can be explained by the successful incorporation of GA into the structure of eMIP. The appearance, elasticity and color properties of the synthesized cryogels were found to be compatible with the literature (He et al. 2021).

Within the scope of characterization results, the surface analyses are compared with the literature, it can be said that eMIPs and NIPs showed that both of them have homogeneously distributed interconnected macropores and cryogels were successfully synthesized (Zenger & Pesint, 2022). Considering the shifts and intensity changes observed in the FTIR spectras are, it can be said that GA and VIM successfully formed a preliminary complex. The changes observed in the spectras in the FTIR plots indicate that the GA-VIM pre-complex is incorporated into the structure of eMIP.

The interaction behavior between adsorption isotherms and cryogel discs and TA molecules was investigated using experimental adsorption data. Adsorption isotherms reveal the relationship between the amount of adsorbate (Qe) adsorbed on the adsorbent and the equilibrium concentration (Ce) of the adsorbate in the solution (Zenger and Peşint 2022).When the obtained R^2^ values ​​are evaluated, it can be argued TA adsorption more fitted with Langmuir isotherm that TA binding sites on the surface of prepared eMIPs are homogeneously distributed, monolayer, equi-energy, and with minimal lateral interaction.

When the release analyses are evaluated, it can be said release rate increases as the initial TA loading amount increases. This is an expected result and can be explained by the increase in the driving force for the diffusion of TA from the cryogel cover materials as the amount of TA in the cryogel cover materials increases. In Table 3, n values are smaller than 0.5. This indicates that the TA is released from the cryogel cover materials by a non-Fickian diffusion mechanism (Çetin and Denizli 2015). Non-Fickian diffusion is often observed in complex systems such as polymeric materials, gel structures or heterogeneous media. Non-Fickian Diffusion is a crucial component in drug delivery systems for understanding and optimizing how the drug release rate changes over time (Çetin and Denizli 2015; Varlık et al. 2023).

Antibacterial activity difference between *S.aureus* and *E.coli* is explained by cell wall structure. Because of having cell walls composed of thick peptidoglycan layer *S. aureus*, TA is rapidly pass through the bacterial cell wall in comparison with *E.coli* and interfere with the internal membrane structure. When the concentration of TA increased there was no significant changes occured on the inhibition zone diameters. An inhibition zone of 12 mm was considered as an effective indicator for strong antimicrobal activity (Hsieh et al. 2001). As a result, it can be concluded that TA loaded cover materials showed effective antimicrobial activity against both *S. aureus* and *E.coli*. Please keep in mind that the TA has toxic effect above the concentration of 5 mg/mL (Deng et al. 2019). According to literature, TA has been shown antimicrobial activities through various Gram (+) and Gram (**−**) microorganisms, such as *S. aureus*, *E. coli*, *Streptococcus pyogenes*, *Enterococcus faecalis*, *Pseudomonas aeruginosa*, *Yersinia enterocolitica*, *Listeria monocytogenes*, *Listeria innocua*, *Bacillus cereus*,* Klebsiella pneumoniae*,* Helicobacter pylori* etc. (Kaczmarek 2020). In a study conducted by Kim et al. tannic acid samples strong antimicrobial activity on *Salmonella typhimurium* and *Enterobacter sakazakii* (Kim et al. 2010). The antimicrobial mechanism of TA could be iron deprivation which may work like a siderophore to chelate essential iron from the medium and make it unavailable to the microorganism (Chung et al. 1998). Moreover, Dong et al. studied the antimicrobial and antibiofilm activity of TA against *S. aureus* and concluded that antimicrobial activity of tannic acid could be attributed with the binding directly with the peptydoglycan layer of the cell wall as a result destroy its integrity (Dong et al. 2018). In 2019, Dabbaghi et al., studied on the synthesis of antibacterial superabsorbents based on tannic acid and resulted that the synthesized material showed antibacterial activity against both *S.aureus* and *E.coli* with being the most effective against *S. aureus* (Dabbaghi et al. 2019).

Cell viability for both materials exhibited no significant change when compared to the 2D control throughout any incubation time. This suggests that neither the substance nor the included tannic acid exhibits cytotoxicity or impedes cellular growth.

## Conclusion

Epitope-imprinted pHEMA-based cryogel cover materials utilizing the antimicrobial effects of TA molecules were produced by using GA as epitope. In line with this objective, GA-imprinted cryogel cover materials were synthesized and GA removed to produce TA recognition sites. TA loading was then performed at different concentrations, and the maximum TA adsorption capacity was determined as 7 mg/mL. TA release studies showed that the high rates of TA release occurred in the first few minutes due to the burst effect, followed by lower rates of release. Antimicrobial properties were investigated on *E.coli* and *S.aureus*, and strong antibacterial effect was observed with no significant difference between gram-positive and gram-negative bacteria. MTT assay results revealed that the synthesized materials exerted no cytotoxic effect on HaCaT cells and promoted cell growth. Cell proliferation was found to be higher in eMIP cryogels compared to NIP cryogels, with slightly higher viability observed in the former. Cell viability for both materials did not significantly differ compared to the 2D control group, indicating no toxic effects of the materials or loaded tannic acid on cells, allowing proliferation to occur unimpeded. As mentioned in discussion section, this study not only contributes a novel biomaterial with significant antimicrobial and wound healing capabilities to the field but also opens avenues for further research on bioactive agent-loaded cryogels in biomedical applications.

## Data Availability

No datasets were generated or analysed during the current study.
